# Dynamic changes in retinal vessel density observed by optical coherence tomography angiography after phacoemulsification: active vs gravity fluidics system

**DOI:** 10.5935/0004-2749.20220093

**Published:** 2025-08-21

**Authors:** Xin Liu, Yanwen Fang, Yao Zhou, Min Wang, Yi Luo

**Affiliations:** 1 Department of Ophthalmology, Eye and ENT Hospital of Fudan University, Shanghai, China; 2 Key NHC Key Laboratory of Myopia (Fudan University); Laboratory of Myopia, Chinese Academy of Medical Sciences, Shanghai, China; 3 Shanghai Key Laboratory of Visual Impairment and Restoration, Fudan University, Shanghai, China

Dear Editor,

During phacoemulsification, the ocular environment changes because of the heat generated by dissipated ultrasound energy, high perfusion, fluctuation in intraocular pressure (IOP), and the inflammatory response resulting from surgical manipulation. These factors may lead to ocular insults, including morphological changes, functional changes, and vascular alteration^(1)^. The active fluidics (AF) system using the Intrepid balanced tip can effectively reduce postocclusion surge and achieve lower cumulative dissipated energy values than the gravity fluidics (GF) platform can, suggesting higher surgical efficiency and better protection^(2)^. Previous studies have demonstrated that macular microcirculation increases after phacoemulsification^(3)^. The fluctuation of vessel density (VD) at the parafoveal region was more significant in the eyes of the GF group than in the AF group^(4)^. However, more detailed dynamic changes between the two systems in the retinal microvasculature after phacoemulsification remain to be explored.

This prospective observational study was performed at the Eye and ENT Hospital of Fudan University, Shanghai, China. Patients with age-related cataract who received phacoemulsification and intraocular lens (IOL) implantation using the Centurion Vision System (Alcon Laboratories Inc.) were enrolled. Twenty consecutive patients (20 eyes) who underwent phacoemulsification with AF were included in the AF group. Another 20 consecutive patients (20 eyes) who underwent phacoemulsification with GF were included in the GF group. The VD was quantified in the whole image and the circumpapillary and parafoveal regions were examined with optical coherence tomography angiography before phacoemulsification, at 1 and 4 hours postoperatively, and every day from 1 to 4 days, 1 week, and 1 month after surgery. All patients provided written informed consent. This study was carried out with the approval of the Institutional Review Board of Eye and ENT Hospital of Fudan University and in accordance with the Declaration of Helsinki.

**Figure 1:**
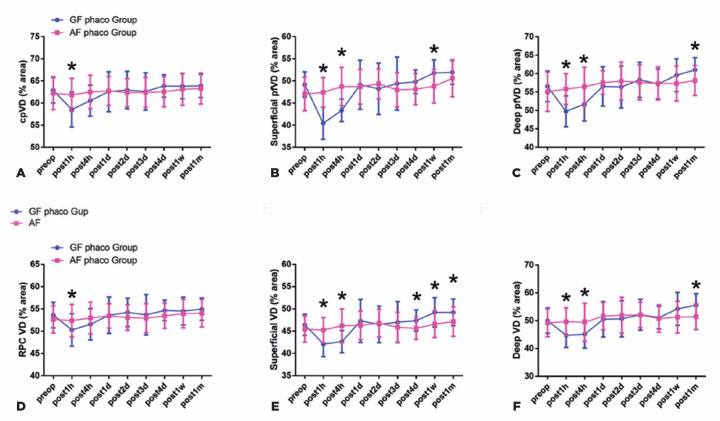
Comparison of the peripapillary and parafoveal microvasculature between the active and gravity fluidics groups. (A, D) In the peripapillary region, the average circumpapillary VD and wiVD values of the AF group were significantly higher than those in GF group 1 hour postoperatively (*p*<0.001 for both). (B, E) In the superficial layer of the parafoveal area, the average pfVD and wiVD values in the AF group were significantly higher than those in the GF group at 1 and 4 hours after the operation (*p*<0.001 for both). The mean pfVD value in the AF group was significantly lower than that in the GF group at 1 w postoperatively (*p*<0.05). The mean wiVD value in the AF group was significantly lower than that in the GF group at 4 days, 1 week, and 1 month after the operation (*p*<0.05). (C, F) In the deep layer of the parafoveal area, the mean values of both the pfVD and wiVD in the AF group were significantly higher at 1 and 4 hours postoperatively and were significantly lower at 1 month after the operation as compared with those in the GF group (*p*<0.05 for all).

**Figure 2:**
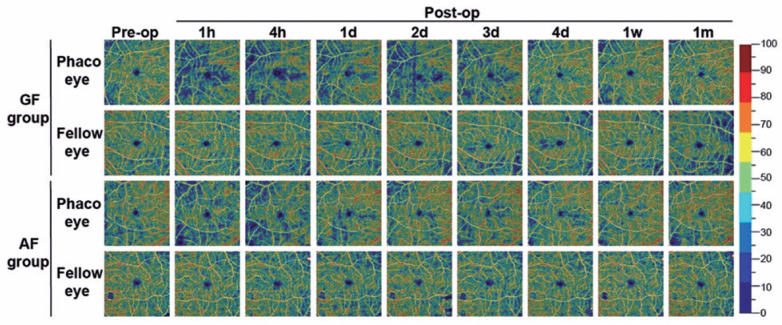
Dynamic change in the superficial layer of the parafoveal microvasculature after phacoemulsification with gravity fluidics compared with active fluidics. The figure displays the whole en face optical coherence tomography angiography images of the superficial layer of the parafoveal (6 × 6 mm) area taken before phacoemulsification, at 1 and 4 hours postoperatively, and each day from 1 to 4 days, 1 week, and 1 month after the surgery, respectively. Images of eyes that underwent phacoemulsification (Phaco eye) and the contralateral unoperated eyes (fellow eye) were taken from patient No. 18 in the GF group and patient No. 16 in the AF group.

The dynamics of the alteration in retinal microvasculature after phacoemulsification between the GF and AF system was evaluated. After surgery, the average circumpapillary VD and the whole en face image VD (wiVD) values in the peripapillary area were significantly higher in the AF group than in the GF group at 1 hour postoperatively (p<0.001; Figure 1). With regard to the VD of the superficial layer in the parafoveal area, the average VD of the parafoveal region (pfVD) and wiVD values in the AF group were significantly higher than those in the GF group at 1 hour and 4 hours postoperatively (p<0.001 for both). The mean pfVD value in the AF group was significantly lower than that in the GF group at 1 week after the operation (p<0.05). The mean wiVD value in the AF group was significantly lower than that in the GF group at 4 days, 1 week, and 1 month postoperatively (p<0.05; Figures 1 and 2). The mean values of both the pfVD and wiVD in the deep layer of the AF group were significantly higher at 1 hour and 4 hours postoperatively and were significantly lower at 1 month after the operation as compared with those in the GF group (p<0.05 for all; Figure 1).

Reasons for the changes in retinal vasculature are not fully clear. Transient high IOP and IOP fluctuation might play major roles. The rapid increase in IOP results in a series of insults to the retinal cell, including the disturbance of retinal blood flow, ischemia reperfusion injury, overload of reactive oxygen species, inflammatory cytokines, and increased vascular permeability^(5)^. These changes may cause damage of vessel autoregulation and fluctuation in blood flow.

Taken together, these results demonstrate that there was a rapid decrease in peripapillary and parafoveal VD 1 hour after the operation, which gradually recovered within 1 week. The VD was much more stable and displayed lower fluctuation after phacoemulsification with the AF system. These findings indicate that the AF configuration offers improved surgical efficiency, with much greater microvascular stability and a better protective effect on the retinal microvasculature.
